# Experiences of being a single young mother caring for children with severe intellectual disability: A qualitative study

**DOI:** 10.4102/curationis.v49i1.2885

**Published:** 2026-10-22

**Authors:** Sylvia M. Malebatsa, Charlene Downing

**Affiliations:** 1 Department of Nursing, Faculty of Health Sciences, University of Johannesburg, Johannesburg, South Africa

**Keywords:** experiences, single young mothers, caregiving, child, disability, severe intellectual disability, support system, South Africa

## Abstract

**Background:**

Single young mothers raising a child with severe intellectual disability face distinct day-to-day challenges. For many, the caregiving role is both demanding and life-changing. Beyond typical parenting duties, mothers often provide intensive care and must navigate health and support systems that can be difficult to understand and access. This can create ongoing uncertainty as they manage daily care and supervision, make sense of complex diagnoses and care needs, cope with emotional and practical demands, and seek appropriate resources and services. As they work to secure suitable care and services for their children, mothers may experience ongoing uncertainty, stress and worry.

**Objectives:**

The study aimed to explore and describe the experiences of single young mothers caring for children with severe intellectual disability.

**Method:**

A qualitative, exploratory and descriptive research design was adopted to capture the nuanced experiences of eight purposefully sampled single young mothers caring for a child with severe intellectual disability.

**Results:**

Three themes emerged: (1) Knowledge gaps among single young mothers, (2) Emotional and psychological impact of the diagnosis and (3) Strength and support systems among single young mothers.

**Conclusion:**

The findings can guide professionals in developing initiatives for single young mothers that strengthen financial stability, overall well-being, and caregiver education in caring for their children.

**Contribution:**

The study provides in-depth insight into the experiences of single young mothers caring for children with severe intellectual disability in the South African context. It highlights the knowledge gap, emotional challenges and support needs. The findings inform contextually relevant recommendations to strengthen professional support, enhance maternal well-being and improve quality of life for both the mothers and their children.

## Introduction

### Background

Caring for a child with severe intellectual disability can place profound psychological and emotional demands on single mothers, affecting mental health, coping, family dynamics and social participation. Parents of children with intellectual disabilities also commonly report heightened stress and emotional distress, alongside an increased caregiving burden that can reduce overall well-being and resilience (Yang 2025).

Single young mothers often need to adapt to major day-to-day changes, including accepting the diagnosis, identifying and accessing support systems, and developing coping strategies to manage caregiving demands (Buthelezi & Mawila 2024). Common challenges include restricted access to essential services, financial hardship, psychological stress, social isolation and stigma, which can negatively affect well-being and life satisfaction (Hadebe 2024; Mbanjwa & Harvey 2023).

Intellectual disability (ID) typically manifests before age 18 and affects an estimated 1%–3% of the general population, with varying degrees of severity. Kimura and Yamazaki (2013) and Brown et al. (2022), drawing on the definition of the American Association on Intellectual and Developmental Disabilities, describe ID as a condition characterised by significant limitations in intellectual functioning and adaptive behaviour that affect everyday social and practical skills. The DSM-5-TR similarly describes ID as an impairment that affects independence, social responsibility and daily living skills, with four severity levels: mild, moderate, severe and profound.

According to the United States Census Bureau (Kramer 2019), the US had the highest rate of children living in single parent households of any nation in the world, at about 23%.,more than in any country (Pew Research Centre Kramer 2019)The number of single mothers living without a partner increased between 2017 and 2022, and 51.4% of single mothers had never been married.

Sartor, Lange and Tröster (2022) estimated that 83% of single mothers across 45 countries globally care for children with disabilities. The United Nations Disability Inclusive Communication Guideline (United Nations 2022) estimates that persons with disabilities constitute about 15% of the world’s population, and that approximately one in ten children has a disability.

In sub-Saharan African countries, the percentage of children living with a single parent ranges from 13% to 43%. In Africa, the prevalence of single motherhood is reported as 50% among never-married women in Southern Africa and 15.7% in Western Africa; among women aged 20–24 years, it reaches 60.7% (Ntoimo & Mutanda 2021).

In South Africa, Samuels, Stimela and Booi (2020) found the absence of relevant and reliable statistics on children with intellectual disability and young mothers; available estimates suggest a prevalence of approximately 11%. Compared with married mothers, single mothers are more likely to live below the poverty line, which is associated with socioeconomic status, educational level, employment status and financial resources (Sadiki, Watermeyer & Abrahams 2021; Seroke & Mkhize 2023). Hadebe (2024) reported that 12% of the population with disabilities have an ID, ranging from mild to profound.

According to Modula (2022) and Brown et al. (2022), South Africa has about 12% of children with intellectual disabilities, with Gauteng Province reporting approximately 5%. This study focuses specifically on severe intellectual disability (SID), characterised by major functional impairments that manifest before age 18 years and an intelligence quotient (IQ) below 20–35.

Studies by Van der Mark et al. (2019) and Mmangaliso and Lupuwana (2021) found that caregivers of children with SID experience higher levels of difficulty than caregivers in non-disability contexts. Commonly reported challenges include stress, isolation, anger, frustration and shame, heightened psychological, physical, financial and relationship strain compared with other mothers (Capri et al. 2018; Hadebe 2024). Single mothers of low socioeconomic status often face elevated stress, financial sstrain, stigma and discrimination (Buthelezi & Mawila 2024).

Single mothers often struggle to obtain support from service providers (Mkabile et al. 2021). Caregivers may also have difficulty accessing appropriate information and care from health professionals to understand their children’s diagnoses (Van der Mescht et al. 2023), with similar findings reported by Hadebe (2024). These ongoing responsibilities can reduce mothers’ social interaction and contribute to isolation.

These responsibilities can also create emotional, financial and social burdens for parents (Mbanjwa & Harvey 2023). Families caring for children with SID may experience poor nutrition and live in socioeconomically disadvantaged areas. Many rely on monthly social grants of approximately R1600 to R2090, and unemployment is frequently the primary route to qualifying for disability grants in the absence of a regular basic income (Modula 2022; Pillay & Siyothula 2011; Van der Mark et al. 2019).

Caring for children with SID in South Africa requires access to specialised knowledge and multiple forms of support (Mbanjwa & Harvey 2023), such as healthcare and ongoing clinical support, appropriate education and developmental support services. Financial assistance and physical and psychological support are required to ease the caregiving burden of caregivers. Studies in several African countries have highlighted how cultural and religious beliefs shape families’ experiences of caring for children with intellectual disabilities (Mazibuko, Ramukumba & Ngwenya 2022; Modula 2022; Raliphaswa, Maluleke & Netshikweta 2022; Tigere & Makhubele 2019; Yang 2025). In some communities, SID may be interpreted as a spiritual condition, and affected families may be viewed as recipients of divine punishment (Dambi, Jelsma & Mlambo 2014; Oti-Boadi 2017; Mbanjwa & Harvey 2023; Mkabile et al. 2021; Seroke & Mkhize 2023; Tigere & Makhubele 2019).

Limited educational programmes may leave parents as unpaid caregivers, which can restrict employment opportunities (Capri et al. 2018; Modula 2022). In addition, there is often minimal coordination between health, education, and social development services to address families’ needs (Modula 2022; Van der Mescht et al. 2023). Strengthening community-based services through task-shifting and improved monitoring may enhance assessment, intervention and referral for people with intellectual disabilities (Ozsavran, Karakaya & Bahadır 2025).

Cultural and spiritual beliefs can shape how ID is understood, with some communities attributing it to witchcraft, curses or divine punishment (Modula 2022; Tigere & Makhubele 2019). These interpretations may influence single mothers’ reactions, the support they seek (including from traditional or spiritual healers), and how they manage their child’s care. Mothers may also experience limited support from family, friends and the wider community, alongside financial constraints and stigma, all of which can negatively affect well-being and overall life satisfaction (Mbanjwa & Harvey 2023; Van der Mark et al. 2019; Yang 2025).

### Problem statement

Caring for a child with SID can result in substantial psychological, emotional, financial and social demands that affect daily functioning (Mkabile et al. 2021). Mothers most often bear the primary responsibility (Modula 2022; Seroke & Mkhize 2023). In many households, single young mothers carry the responsibilities alone, experiencing daily hardships with limited access to support services (Mmangaliso & Lupuwana 2021; Yang 2025). The pressure can negatively affect the maternal well-being and daily functioning. Although previous research has explored the experiences of parents and mothers caring for children with intellectual disabilities, limited attention has been given to the specific experiences of single young mothers caring for children with SID, particularly within the South African context, particularly in Gauteng Province. Much of the available literature looks at caregivers as homogenous, failing to account for age, single motherhood, socioeconomic disadvantage and limited access to services that can assist in shaping caregiving. Without a deeper understanding of these single mothers’ challenges, coping strategies and support needs, and existing social services remain a challenge that affects the daily realities. The gap highlights the need to explore experiences of single young mothers and to generate insights, enhance maternal well-being and help empower single young mothers to manage everyday caregiving responsibilities in the Gauteng Province.

## Research methods and design

### Study design

A qualitative, descriptive and exploratory research design was used for this study. The research design was formulated to gain an in-depth understanding of the experiences of single young mothers caring for their children with SID. An exploratory design was used to examine an under-researched phenomenon within the South African context, while the descriptive component enabled a detailed account of participants’ experiences (Polit & Beck 2020). Individual semi-structured interviews were conducted to collect data (Creswell & Creswell 2023; Leavy 2017).

### Setting

The study was conducted in Gauteng, City of Johannesburg, Metropolitan Municipalities (region E and region A [including parts of Alexandra, Marlboro and Ivory Park, part of Midrand]). Participants’ recruitment was facilitated through Nokuthula Special School in Lyndhurst, where single young mothers’ children attend school.

Interviews were held at venues selected by participants, such as participants’ homes. These areas are characterised by predominantly urban and peri-urban environments with marked socioeconomic disparities. The setting was chosen for its diverse population and availability of households of single young mothers caring for children with SID. Access to participants was facilitated through Nokuthula Special School in Lyndhurst, which educationally supports families of children with disabilities.

### Study population and sampling strategy

The study population comprised single mothers in Gauteng Province who were caring for a child with SID (Kviz 2020). A population refers to the entire group of interest that meets the study criteria (LoBiondo-Wood & Haber 2021; Willie 2024). For this study, the target population included single young mothers aged 18–35 caring for a child with SID in Gauteng Province. The accessible population consisted of mothers who met these criteria and voluntarily consented to participate in the study.

Although 14–16 interviews were initially planned, data saturation was reached after eight participants, with no additional themes emerging. Data saturation occurs when no significant new information emerges (Polit & Beck 2020). The primary aim was to reveal patterns, categories, and dimensions of the phenomenon under investigation. Data saturation refers to the point at which collecting additional qualitative data yields no new information (Korstjens & Moser 2018). LoBiondo-Wood and Haber (2021) describe data saturation as the process of obtaining the full range of themes from participants until no new data emerge. The researcher interviewed eight participants, and sufficient, detailed data were collected and described; information was considered redundant when further sampling provided no new insights (Gray & Grove 2021).

Purposive sampling was used to deliberately select participants who had direct experience and were able to provide rich, in-depth information about the phenomenon under study (Creswell & Creswell 2023; Polit & Beck 2020; Shaheen, Pradhan & Ranajee 2019). This sampling approach is appropriate for this qualitative research aimed at exploring the lived experiences of single young mothers (LoBiondo-Wood & Haber 2021). Recruitment was supplemented through snowball sampling, whereby participant referrals helped to identify additional eligible participants (Trochim, Donnelly & Arora 2016). Recruitment was facilitated through Nokuthula Special School in Lyndhurst, which supports families of children with intellectual disabilities. Eight single young mothers participated in the study. Although an initial sample size of 14–16 participants was anticipated, data saturation was reached after eight interviews, as no new themes emerged during data analysis. The final sample size was therefore considered sufficient to achieve the study objectives and ensure depth and credibility of the findings.

### Inclusion and exclusion criteria

#### Inclusion criteria

This study applied predetermined inclusion criteria to ensure that participants possessed the characteristics needed to address the research question (Grey & Grove 2021; Pyo et al. 2023). Inclusion criteria are the specific attributes prospective participants must meet to be eligible for participation (Creswell & Creswell 2023; Pyo et al. 2023). Participants were eligible for inclusion in the study if they met all of the following criteria: Were single young mothers who had never been married, were between 18 and 35 years of age, and were caring for a child with SID in Gauteng Province.

Participants who met these criteria were purposively selected, as they were well-positioned to provide rich, relevant descriptions of the phenomenon under investigation. This approach strengthened the relevance and quality of the data collected.

#### Exclusion criteria

Exclusion criteria were applied to identify individuals who did not meet the requirements for participation and who could potentially compromise the focus of the study (Patino & Ferreira 2018). Some participants who were recruited through Nokuthula Special School in Gauteng were excluded as they did not meet a specified age range of 18–35, married, divorced or widowed.

### Data collection

Data were collected through individual semi-structured interviews, allowing participants to share their personal narratives in depth (Flick 2020). The researcher utilised an interview guide, developed with the guidance of the research supervisor. The guide was designed in English to align with the main research question and objectives. The interview guide included open-ended questions designed to elicit detailed accounts of daily caregiving responsibilities, challenges encountered, sources of support and coping strategies. All interviews were conducted in a convenient and comfortable setting to foster participant trust. Confidentiality was maintained by de-identifying names. With participants’ consent, field notes and audio recordings were collected. The interview questions were adapted to ensure that participants’ perspectives were acknowledged, and their distinct experiences were authentically documented. Interviews were conducted in isiZulu, Setswana, Sepedi or English, depending on each participant’s language preference.

The purpose of the study was explained in writing, and verbal consent was obtained. Interviews were the primary method of data collection, guided by a semi-structured protocol with open-ended questions. Participants were informed that their data could be published in scientific journals, that interviews would be recorded, and that they could withdraw from the study at any time without facing consequences. Participation was voluntary, in line with ethical guidelines (Budak, Küçük & Civelek 2018; DePoy 2024). Interviews were held in isiZulu, Sepedi, Tswana and Xitsonga, the participants’ native languages, which were translated and transcribed into English, and lasted between 45 and 60 min. The translated transcripts were verified with the participants.

### Data analysis

Thematic analysis centred on the main research question: ‘What are the experiences of being a single young mother with a child with severe intellectual disability?’ This method identified and reported patterns in data through a six-step process: familiarisation, coding, theme development, evaluation, definition and final report (Braun & Clarke 2014). The researcher used field notes, listened to recordings and transcribed eight interviews verbatim, verifying accuracy by reviewing recordings multiple times. The audio recordings and transcripts were sent to the research supervisor for review, ensuring all interview details were accurately documented. Personal information was anonymised during transcription, and each participant received a unique code to maintain confidentiality. Throughout the study, the researcher followed established ethical guidelines, such as obtaining informed consent, honouring participants’ autonomy, and clearly outlining the study’s aims and the potential publication of results. The researcher’s reflexivity and continuous awareness of power dynamics further supported the ethical standards and integrity of the research.

The researcher reviewed and coded the transcripts using inductive coding. Initially, codes were organised into major themes. According to Braun and Clarke (2021), thematic analysis involves a structured approach with guided coding and theme development. The transcripts were interactively examined, codes were manually created, and patterns were identified and sorted into themes and subthemes. A pilot interview was conducted and included in the final dataset.

### Trustworthiness

To ensure trustworthiness, the study adhered to the criteria of credibility, transferability, dependability and confirmability (Lincoln & Guba 1985). Credibility was achieved through prolonged engagement with participants, member checking and triangulation of data sources. Field notes, audio recording and interview transcripts were included. The use of verbatim transcription and supervisor review further strengthened the authenticity of the findings. Transferability was enhanced by providing rich, contextual descriptions of the research setting and participant characteristics, allowing readers to determine the applicability of the findings to other contexts. For dependability, the researcher maintained a comprehensive audit trail documenting methodological decisions and changes throughout the research process. Confirmability was ensured by reflexive journaling and maintaining transparency in data analysis, minimising potential researcher bias and ensuring the results reflected participants’ experiences as accurately as possible while maintaining confidentiality and anonymity (Creswell & Creswell 2023).

### Ethical considerations

This study was approved by the University of Johannesburg’s Faculty of Health Sciences, Department of Nursing, Higher Degrees Committee and Research Ethics Committee (REC-2307-2023), and the Department of Higher Education review board (HDC-HDC-01-72-2023). Ethics approval was also granted by the Gauteng Department of Education Research Ethics Committee (2024/316) and Gauteng Department of Health Research Committee (NHRD REF. NO: GP_202408_006). Ethical principles guided the study: autonomy, beneficence, non-maleficence and justice. Informed consent was secured from all participants before data collection.

## Results

Three themes and eight subthemes were identified (see Table 1).

**TABLE 1:** Themes and subthemes.

Themes	Subthemes
1. Knowledge gap experienced by single mothers	1.1. A lack of awareness about the condition
2. Emotional and psychological impact of diagnosis	2.1. Mother’s experience of regret 2.2. Experience of maternal fear and worry 2.3. Experiences of stigma and judgement
3. Strength and support system	3.1. Internal support system 3.2. External support system 3.3. Admission to special school 3.4. Financial constraints

Table 2 shows the demographic population characteristics of the sample.

**TABLE 2:** Study population demographic characteristics.

Participant	Age (years)	Language	Educational background	Geographical area	Employment status	Age of the child (years)
1	24	English, Zulu, Tswana	Grade 11	Gauteng	Unemployed	7
2	32	Tsonga, Sepedi	Grade 9	Gauteng	Assistant teacher	16
3	32	English, Tsonga, Sepedi	Grade 12	Gauteng	Assistant teacher	6
4	34	Tsonga, Sepedi	Grade 9	Gauteng	Unemployed	16
5	29	Sotho	Grade 8	Gauteng	Unemployed	9
6	32	English, Zulu	Grade 12	Gauteng	Unemployed	9
7	32	Tsonga, Zulu	Grade 12	Gauteng	Unemployed	9
8	35	Sepedi, Zulu	Grade 12	Gauteng	Temporary employment	6

Three overarching themes with associated subthemes emerged from the thematic analysis, capturing the lived experiences of single young mothers caring for children with SID. These themes reflect participants’ knowledge gaps, emotional and psychological responses to diagnosis, and the support systems they relied on in their daily lives.

### Theme 1: Knowledge gaps experienced by single mothers

This theme highlights the limited knowledge and understanding mothers had regarding SID, particularly during the early stages of caregiving and diagnosis. Most participants reported difficulty in recognising early signs of developmental delay and expressed confusion and distress when attempting to understand their child’s diagnosis.

Mothers described the diagnostic process as poorly explained and characterised by limited communication from healthcare professionals. The lack of clear, respectful explanations contributed to fear, uncertainty, and emotional distress.

#### Subtheme 1.1: A lack of awareness of the condition

Many participants reported that their children initially appeared healthy, and developmental delays were not immediately recognised as signs of disability. Mothers often attributed delays in sitting, walking, or speech to individual variation rather than possible ID, partly because of limited prior exposure to disability:

‘I did not understand my child’s diagnosis. The person explaining was impatient. I was scared to ask more questions.’ (Participant 7, 32 years old, unemployed)

The absence of visible physical abnormalities further delayed recognition and understanding of the condition:

‘My child was born normal. He was born like other children, with no abnormalities or disabilities … No one could have suspected that he was intellectually disabled by looking at him because he looked clever, with no indication.’ (Participant 6, 32 years old, unemployed)

The absence of visible physical signs delayed recognition of the condition:

‘He then started repeating words when he talked and would sometimes play by the corner by himself. I did not understand. I thought he was shy. He was sometimes hyperactive, but I thought he was just being a child. I took him to the clinic several times; however, I could not understand what was wrong. I finally received a diagnosis that my child is autistic when he was 7 years old. The teacher referred my son to the Department of Education for assessment. That is how I received the diagnosis.’ (Participant 6, 32 years old, unemployed)

### Theme 2: Emotional and psychological impact of diagnosis

This theme explores how receiving a diagnosis negatively impacted the mother emotionally and psychologically. For many participants, the diagnostic process was unsettling and was associated with shock, disbelief, denial, fear and distress.

The diagnosis altered mothers’ expectations for their children’s futures and intensified their caregiving responsibilities, resulting in heightened emotional burden:

‘Since my child was born normal, with no physical disabilities, I did not want to believe although there were signs of behavioural abnormalities. that there was a problem with my child, it came as a shock.’ (Participant 3, 32 years old, assistant teacher)

‘I sometimes think I should die since my son is 16 years old but still wearing a nappy and cannot bath himself. At the same time, I fear that both my children will suffer in my absence. it really stresses me every day that I would just burst into tears and have sleepless night. I am tired.’ (Participant 4, 34 years old, unemployed)

#### Subtheme 2.1: Mothers’ experience of regret

When a child is diagnosed with a SID, the experience of parenthood is fundamentally altered, and expectations about the child and the future may need to be reassessed (Tigere & Makhubele 2019). Mbanjwa and Harvey (2023) described reactions of shock, frustration, and self-blame to the initial diagnosis, highlighting the stress involved. Most mothers initially seek answers to questions about the nature of the child’s disability from doctors, whose explanations are often unclear.

Participants were required to anticipate their child’s needs, behaviours, and progressive decline. They reported limited social engagement and frequently described living in isolation from broader society:

‘I sometimes regret not knowing that I was pregnant with a child who would get me into trouble. If I knew that she would be normal like other children, I would have terminated the pregnancy. She needs to be watched at all times; I fear that she will be hurt by the community.’ (Particpant 8, 35 years old, temporarily employed)

‘I initially took my son to one of the public hospital and was not given the correct doses of medication. I regret everyday because My son got worse and kept on falling all the time he has got bruises in the forehead, as you can see. He sonly got better when I consulted with the neurologist in Limpopo.’ (Participant 7, 32 years old, unemployed)

Caring for a child with a disability presents challenges for families. It requires significant adjustments in daily life (Shahali et al. 2024). Caregiving can have various effects on the caregiver’s health. Tigere and Makhubele (2019), Van der Mescht et al. (2023) stated that caregivers are expected to cope with the child’s disruptive symptoms or behavioural changes. These changes lead to modifications in household routines (dynamics) and the quality of care. Challenges may also affect the educational, social and emotional needs of the child with disability, which puts the mother under a lot of strain.

#### Subtheme 2.2: Experiance of maternal fear and worry

Ongoing fear and worry were common among participants as they devoted much of their lives to caregiving. They reported persistent concerns about their own ability to cope and concerns about their children’s future:

‘I fear having another child. What if I give birth to another child who has the same condition? “I won’t cope because I am working at the same time.” I ask myself questions like Who will look after my children if I die.’ (Participant 3, 32 years old, unemployed)

*A second participant expressed her fear and worries as follows*:

‘I had questions for myself and God. I asked, “Why, God, why did you give me a child like this one?” I sometimes get very emotional, lock myself in, and cry and also yell at my children for no reason. I also worry a lot about my son’s future.’ (Partcipant 1, 24 years old, unemployed)

#### Subtheme 2.3: Experiences of stigma and judgement

Many mothers reported ongoing distress as a result of social stigma, including mockery, gossip, blame and exclusion within the families and communities. Such experiences occurred in public spaces, institutions, families and social circles, often leading to shame, isolation and emotional strain:

‘My child and I were labelled and mocked because he could not speak well. The neighbours mentioned that his condition was a result of my mother’s witchcraft. At the same time, my own brother and my mother were saying it is a punishment from God of something wrong I practised.’ (Participant 3, 32 years old, assistant teacher)

‘I don’t know my parents. My mother left me when I was still young. My neighbours always say my child is unable to speak and behaves the way he does because I was left alone when I was a child. We don’t have a family; we are always staying in the room, even during holidays.’ (Partcipant 5, 29 years old, unemployed)

### Theme 3: Strength and support system

A significant proportion of participants in this study were either unemployed or underemployed and heavily reliant on social grants, which often fell short of covering the full spectrum of their children’s needs. Despite the many challenges they faced, participants demonstrated resilience through various sources of support. These support systems were essential in helping mothers to manage caregiving responsibilities and cope with emotional and practical demands.

Most participants and their children faced shortages of necessities and living conditions characterised by inadequate nutrition. They lived in unsuitable housing with poor sanitation facilities and experienced financial hardships worsened by unemployment (Zovko, Šimleša & Olujić Tomazin 2024). Mothers often navigate various processes to access essential resources (Mbanjwa & Harvey 2023).

#### Subtheme 3.1: Internal support system

Despite challenges, mothers highlighted essential support from family members, including parents, siblings and extended relatives. That kind of support was regarded as a key source of emotional and primary assistance in easing the burden of caregiving, and faith groups in meeting caregiving demands:

‘During school holidays, the children visit their paternal grandparents.’

‘My family, especially my parents, supports me emotionally and helps me with my child … My parents accompany me to my daughter’s medical check-up once a month.’ (Participant 2, 32 years old, assistant teacher)

A second participant stated:

‘My siblings, aunts, and grandparents are also actively involved in caregiving and providing emotional support.’ (Participant 3, 32 years old, unemployed)

Peer support from other mothers caring for children with disabilities was also important, providing shared understanding and emotional relief. These shared initiatives helped to connect formal services with the realities of everyday caregiving, offering mothers empowerment and reassurance that they were supported throughout their journey:

‘I have got one friend who does not stay far from here, she would ask me to bring my child to care for him, for me to rest or to go to the shops.’ (Participant 4, 34 years old, unemployed)

Participant 6, 32 years old mother, shared the same positive sentiment as follows:

‘When I take my children to the bus in the morning, we meet with the mothers of the children and share our feelings and experiences … After talking with the mothers, I feel relieved and able to carry on with daily life.’ (Participant 6, 32 years old, unemployed)

#### Subtheme 3.2: External support system

**Faith community:** External support included faith-based organisations, schools and community networks. Many mothers described reliance on spiritual beliefs, prayer and church support as important coping mechanisms that provided hope and emotional strength (Van der Mark et al. 2019). These institutions are recognised for providing essential care, love, support, protection and guidance (Tigere & Makhubele 2019).

Churches, pastors and prayer provided valuable support, especially during periods of uncertainty and emotional distress. This is a positive reflection by one participant:

‘“The church, however, supports me, but there is nothing I can do with my child’s condition. It is God’s will.” I am a Christian, and I pray a lot. I get strength from the prayer.’ (Participant 2, 32 years old, assistant teacher)

‘I surrender all to God; He is my refuge. I pray every night, especially for my son. I also thank God for protecting my son at school and around the community.’ (Participant 5, 29 years old, unemployed)

‘I learned to accept and understand that a child can be born normally and change at any stage in life. I did not have the knowledge and understanding of the condition before.’ (Participant 3, 32 years old, assistant teacher)

‘I also accepted the condition since my child started attending school … There are children in wheelchairs. At first, I asked myself why God gave me a child like this one.’ (Participant 2, 32 years old, assistant teacher)

These stories illustrate that acceptance is a fluid process, enabled by access to education, peer support and faith, fostering resilience among mothers (Sakwape, Machailo & Koen 2023).

**Admission to the special school:** Admission to school for children with SID presented significant challenges. As observed by Hadebe (2024), access to special schools is not straightforward, often resulting in mothers waiting 2 to 2 years for their children’s placement. Children who remained at home for extended periods further increased parental responsibilities (Seroke & Mkhize 2023; Van der Mark et al. 2019).

In South Africa, many children with intellectual disabilities face barriers to compulsory schooling because of unclear inclusive education policies (Capri et al. 2018; McKenzie 2020). The White Paper on the Rights of Persons with Disabilities (South Africa 2016) now advocates enrolling all children, regardless of the severity of their disability. While some mothers waited up to 2 years for admission, others benefitted from quicker placement through recommendations. Admissions followed positive assessments by the Department of Education, bringing families optimism and relief. Support came from personal contacts, the Department of Education, and healthcare professionals:

‘The Department of Education found a special school for my child … After two months of application, the school phoned me to say that the application for my daughter was successful.’ (Participant 1, 24 years old, unemployed)

‘When my child is at school, I could improve access to employment. I am very happy.’ (Participant 3, 32 years old, unemployed)

#### Subtheme 3.3: Financial constraints

Financial strain was a persistent challenge for most participants. Unemployment, reliance on social grants, and increased caregiving expenses limited mothers’ ability to meet their children’s specialised needs. Although disability grants provided some assistance, they were widely viewed as insufficient to address ongoing financial demands.

Multiple participants described travelling to clinics and hospitals every 3 months for medical care, often receiving care only from nurses rather than specialists. In addition, they are dependent on government assistance to meet their children’s medical needs (Mmangaliso & Lupuwana 2021):

‘I had to travel with the taxis to the clinic every month without finding a doctor. The clinic was far from home. I used a lot of money that I did not have. It was really hard because sometimes I had to ask for money from other people.’ (Participant 2, 32 years old assistant teacher)

‘Social grant was insufficient to cover the family’s fundamental requirements, and she had not received any financial assistance from the child’s father.’ (Partcipant 5, 29 years old, unemployed)

## Discussion

This study explored the experiences of single young mothers caring for children with SID in Gauteng Province, South Africa. Three interrelated themes emerged from the findings: knowledge gaps regarding SID, the emotional and psychological impact of the diagnosis, and the role of strength and support systems in coping with caregiving demands. This discussion situates these findings within the existing body of literature and highlights their implications for practice and policy.

The findings indicate that many mothers experienced substantial knowledge gaps, particularly during the early stages of recognising developmental delays and receiving a formal diagnosis. Participants described difficulties in understanding medical explanations and navigating fragmented health and support systems, which contributed to confusion, anxiety, and delayed understanding of their child’s condition.

Consistent with previous South African and international studies, caregivers of children with intellectual disabilities often report limited access to clear, timely and comprehensible information from healthcare professionals (Modula 2022; Van der Mescht et al. 2023). Similar challenges were documented by Mbanjwa and Harvey (2023), who reported that single mothers frequently struggle to obtain accurate information and guidance from service providers. Inadequate communication at the point of diagnosis may hinder informed decision-making and negatively affect caregivers’ confidence and coping capacity.

These findings underscore the need for healthcare professionals to prioritise effective, respectful, and culturally sensitive communication. Providing clear explanations, opportunities for questions, and guidance on care pathways at diagnosis may reduce emotional distress and empower mothers to engage more confidently in caregiving roles, as emphasised by Thomas et al. (2024).

The diagnosis of SID had a profound emotional and psychological impact on participants. Mothers described feelings of shock, fear, sadness, regret, and ongoing worry, particularly concerning their children’s futures and their ability to manage long-term caregiving responsibilities. These emotional responses contributed to heightened psychological strain and shaped mothers’ adjustment to caregiving roles (Modula 2022; Sakwape 2022; Yang 2025).

These findings align with existing literature demonstrating that caregivers of children with SID experience significant emotional distress and psychological burden (Hadebe 2024; Van der Mark et al. 2019). Feelings of regret and self-blame reported by participants mirror those identified in studies conducted in other African contexts, where mothers often internalise responsibility for their child’s condition (Oti-Boadi 2017; Tigere & Makhubele 2019).

Social stigma and judgement further compounded emotional distress. Mothers described experiences of blame, gossip, and exclusion within families and communities, which intensified feelings of isolation and vulnerability. These findings are consistent with research highlighting how stigma surrounding disability negatively affects caregivers’ mental well-being and limits their access to social support (Mbanjwa & Harvey 2023; Mkabile et al. 2021).

Despite considerable challenges, participants demonstrated resilience through various internal and external support systems (Sartor et al. 2022). Family members, particularly parents and siblings, played a critical role in providing emotional reassurance, shared caregiving responsibilities and practical assistance (Raliphaswa et al. 2022; Sakwape et al. 2023). Peer support from other mothers raising children with disabilities offered validation and a sense of shared experience (Seroke and Mkhize 2023).

These findings support existing evidence identifying social support as a key protective factor for caregivers of children with disabilities (Sakwape et al. 2023; Zovko et al. 2024). Informal social networks may reduce stress, enhance coping strategies, and promote emotional well-being. Faith-based support also emerged as an important coping resource. Reliance on prayer and spiritual beliefs helped mothers to find meaning, hope and emotional comfort in the face of ongoing challenges. Similar findings have been reported in African studies, where spirituality plays a central role in caregivers’ coping and acceptance of disability (Oti-Boadi 2017; Van der Mark et al. 2019). School enrolment for children with intellectual disabilities requires a comprehensive evaluation by qualified medical practitioners, psychologists or social workers. Studies by Thomas et al. (2024), Modula (2022) and Van der Mescht et al. (2023) similarly report that caregivers of children with intellectual disabilities experience emotional strain and reduced quality of life.

Nevertheless, financial constraints remained a persistent challenge. Most participants were unemployed and dependent on social grants that were insufficient to meet their children’s complex and ongoing needs. This finding reflects previous South African research highlighting the inadequacy of disability grants for families caring for children with SID (Raliphaswa et al. 2022; Seroke & Mkhize 2023).

Modula (2022), as cited by Van der Mescht et al. (2023), together with findings from the South African Social Security Agency (SASSA 2020), further demonstrate that families caring for children with disabilities frequently experience financial strain because of insufficient social or disability grants to meet essential needs. While children with disabilities in South Africa receive a disability grant, this study indicates that it does not fully address their specialised needs. Research in Limpopo reveals that SASSA grants provide essential financial support but may not cover all basic needs of families with children with disability (Raliphaswa et al. 2022; Sakwape et al. 2023). Seroke and Mkhize (2023) also noted that care dependency grants provided to mothers are often insufficient, as they may need to support the entire household. The Department of Basic Education provides for the enrolment of learners with special educational needs, generally from approximately 3 to 21 years of age, in public special schools where appropriate. South Africa’s legislative and policy framework, particularly the Constitution of the Republic of South Africa from 1996, as seen in the Education White Paper 6: Special needs education: Building an inclusive education and training System (Department of Education 2001), and the White Paper on the Rights of Persons with Disabilities (Republic of South Africa 2015), protects the rights, dignity, equality, and access to education of persons with disabilities. Despite these protections, further efforts are necessary to advance the qualification, employment prospects and integration of persons with disabilities within society. To bridge these gaps, systemic reforms combined with individual dedication are necessary. Health communicators, educators, and service providers need current knowledge and strong interpersonal skills to build trust and facilitate understanding. This enables families to overcome confusion and uncertainty, fostering resilience, empowerment and authentic collaboration. These gaps leave mothers not only unsupported but also invisible, reinforcing cycles of isolation, emotional hardship, and financial strain.

### Limitations

The evidence based on single young mothers caring for children with SID remains limited internationally and in South Africa. This study, therefore, focused on single young mothers in Gauteng Province (South Africa) who were caring for a child with SID.

The sample was small; the findings should not be generalised to broader populations, which may limit transferability. However, the sample size was sufficient to reach data saturation, with no new themes emerging as data collection progressed. Further studies should include single young mothers in other South African provinces to strengthen the evidence base and allow for comparisons across contexts.

#### Strength

This study used a qualitative approach to explore and describe the experiences of single young mothers caring for children with SID. The researcher engaged with participants through scheduled appointments until data saturation was reached, which strengthened the depth and richness of the data.

The information letter was discussed in detail to ensure participants’ understanding, and informed consent was obtained before interviews. Participants’ confidentiality and privacy were protected by omitting real names.

The researcher’s age supported rapport-building and helped participants share their experiences openly. To promote clear communication, the researcher used multiple languages and adapted communication techniques when participants could not understand or speak English. Prolonged engagement supported rich, contextual descriptions of participants’ lived experiences. Transcripts were verified through member checking to strengthen credibility.

### Recommendations

The following recommendations are for policymakers.

#### Implications for practice and policy

The findings highlight the need for coordinated multidisciplinary support for single young mothers caring for children with SID. Healthcare professionals should prioritise clear communication, caregiver education, and emotional support at the point of diagnosis. Strengthening collaboration across health, education, and social development sectors may improve access to information, psychosocial support and financial assistance, thereby enhancing maternal well-being and caregiving outcomes. The implementation of policy-informed interventions may contribute to improving access to resources and support for single young mothers caring for children with SID.

### Nursing research

This study contributes valuable qualitative evidence on the experiences of single young mothers caring for children with SID in the South African context. The findings highlight important gaps in knowledge, emotional support, and access to services, and therefore underscore several priorities for future nursing research. More research is needed to explore the informational needs of single young mothers at different stages of their caregiving journey, particularly at the point of diagnosis. Nursing research should also examine how communication practices by healthcare professionals influence mothers’ understanding, emotional responses, and engagement with care. Investigating effective nurse-led communication and education interventions may inform strategies that improve caregiver empowerment and reduce psychological distress. Given the financial constraints experienced by single young mothers, further nursing research should also investigate the intersections between socioeconomic vulnerability, caregiving burden and health outcomes. Overall, nursing research plays a crucial role in generating evidence recommendation of responsive caring models related to this study.

#### Healthcare providers

Nurses may assist the single young mothers by working collaboratively with medical practitioners and social workers by providing respectful, understandable explanations of the child’s diagnosis, prognosis, and care needs.

It is also significant to offer caregiver education on daily care, behaviour management, safety, and stress management and encourage referral and follow-up pathways to mental health services, community programmes, and special education support.

### Education and awareness

Education and awareness initiatives should equip mothers with accurate, practical information about their child’s disability, prognosis and tailored care needs. Offer regular workshops and mentorship opportunities for mothers and encourage interprofessional collaboration to support holistic care, to ensure quality care is supported by adequate resources (financial, human, and material). These findings can be used to inform community programmes that strengthen professional training for healthcare providers and support single young mothers through contextually relevant interventions to reduce stigma and discrimination affecting mothers and their children in South Africa. The social support systems highlighted in the findings suggest peer support groups, community-based initiatives and faith-sensitive care approaches.

## Conclusion

The findings from this study indicate that single young mothers living with a child with SID experience substantial levels of stress. Most mothers experience significant financial strain, physical exhaustion, and an overwhelming daily caregiving burden, alongside heightened anxiety, discrimination by the community, a lack of social support and persistent social stigma. Given these findings, it is recommended that targeted psychosocial interventions, such as counselling services and peer support groups, be prioritised to address the emotional and mental health needs of single mothers caring for children with SID. In addition, partnerships with community organisations and local advocacy groups can play a significant role in reducing stigma and fostering inclusive environments, ultimately promoting resilience and empowerment among these mothers.

The findings highlight the urgent need for coordinated multidisciplinary support for single young mothers caring for children with SID. Healthcare professionals should prioritise clear communication, caregiver education, and emotional support at the point of diagnosis. Strengthening collaboration between health, education, and social services may improve access to information, psychosocial support and financial assistance. The significant emotional and psychological challenges identified in this study point to the need for nursing research focused on mental health outcomes among caregivers of children with SID. Future studies should explore the prevalence of anxiety, depression, and caregiver burnout among single young mothers, as well as the effectiveness of psychosocial support interventions led or coordinated by nurses. Longitudinal research designs may be particularly valuable in understanding how emotional well-being evolves over time and in response to caregiving demands.
